# In Vitro Characterization of Dental Pulp Stem Cells Cultured in Two Microsphere-Forming Culture Plates

**DOI:** 10.3390/jcm9010242

**Published:** 2020-01-16

**Authors:** Nam-Ung Bu, Hyo-Seol Lee, Bin-Na Lee, Yun-Chan Hwang, Sun-Young Kim, Seok Woo Chang, Kyoung-Kyu Choi, Duck-Su Kim, Ji-Hyun Jang

**Affiliations:** 1 Department of Conservative Dentistry, Graduate School, Kyung Hee University, Seoul 02447, Korea; 2 Department of Pediatric Dentistry, School of Dentistry, Kyung Hee University, Seoul 02447, Korea; 3 Department of Conservative Dentistry, School of Dentistry, Dental Science Research Institute, Chonnam National University, Gwang-ju 61186, Korea; 4 Department of Conservative Dentistry and Dental Research Institute, School of Dentistry, Seoul National University, Seoul 03080, Korea; 5 Department of Conservative Dentistry, School of Dentistry, Kyung Hee University, Seoul 02447, Korea

**Keywords:** culture method, dental pulp, dental pulp stem cell, microsphere, multilineage differentiation, pulp regeneration, RNA sequencing

## Abstract

Various three-dimensional (3D) culture methods have been introduced to overcome the limitations of in vitro culture and mimic in vivo conditions. This study aimed to evaluate two microsphere-forming culture methods and a monolayer culture method. We evaluated cell morphology, viability, osteo-, adipo-, and chondrogenic differentiation potential of dental pulp stem cells (DPSCs) cultured in 3D culture plates: ultra-low attachment (ULA) and U-bottomed StemFit 3D (SF) plates, and a two-dimensional (2D) monolayer plate. RNA sequencing (RNA-seq) revealed differentially expressed gene (DEG) profiles of the DPSCs. In contrast to an increasing pattern in the 2D group, cell viability in 3D groups (ULA and SF) showed a decreasing pattern; however, high multilineage differentiation was observed in both the 3D groups. RNA-seq showed significantly overexpressed gene ontology categories including angiogenesis, cell migration, differentiation, and proliferation in the 3D groups. Hierarchical clustering analysis revealed a similar DEG regulation pattern between the 3D groups; however, a comparatively different DEG was observed between the 2D and 3D groups. Taken together, this study shows that DPSCs cultured in microsphere-forming plates present superior multilineage differentiation capacities and demonstrate higher DEG expression in regeneration-related gene categories compared to that in DPSCs cultured in a conventional monolayer plate.

## 1. Introduction

Dental pulp stem cells (DPSCs) extracted from the pulp of human molars are considered a superior source of multipotent mesenchymal stem cells (MSCs) for use in tissue engineering [1]. DPSCs typically express the STRO-1 and CD146 antigens and are able to differentiate into neurons, cardiomyocytes, chondrocytes, osteoblasts, liver cells, and β cells of islet of pancreas [2,3]. As DPSCs can be easily obtained, they have attracted considerable interest on account of their wide potential for use in regenerative endodontics [4]. 

Much of our understanding of the biological mechanisms underlying cellular functions of DPSCs, such as differentiation and multipotency, has been shaped from studying cells cultured on two-dimensional (2D) monolayer dish surfaces [5]. However, recent studies highlight that cells grown on 2D substrates show a simplified morphology and changes in properties, and such changes result in conditions that greatly differ from those of the natural microenvironment [6]. Another study reported that the 2D culture method has limitations because it does not replicate the cell–cell and cell–extracellular matrix (ECM) interactions that tissues possess [7].

Cells, signals, and scaffolds, which are known as the tissue engineering triad, are needed in three-dimensional (3D) tissue regeneration [8]. The culture environment is important because it not only supports cell survival but also provides optimal conditions for the synthesis of the matrix [9]. Various 3D culture methods have been developed to overcome these limitations, which include the hanging drop method, spontaneous spheroid formation, suspension culture, scaffold-based models, and magnetic levitation [10]. The “spontaneous spheroid formation” method is one of the scaffold-free culture methods. In this method, plates coated with an inert substrate, such as agar or poly-2-hydroxyethyl methacrylate (poly-HEMA), are used, thus resulting in cell microspheroids without scaffolds mimicking the physiological cell culture conditions. Poly-HEMA prevents cells from attaching to the surface of the plates, forcing the cells to aggregate and form spheroids. This method is convenient to use because it allows the use of pre-coated plates sold commercially, and thus a high-throughput culture of spheroids [10]. 

Previous studies have described the differences between the monolayer and spheroid culture methods. Baharvand et al. [11] examined the differentiating potential of human embryonic stem cells into hepatocytes in 2D and 3D culture systems by evaluating several cellular characteristics of the hepatocytes, including expression of α-1-antitrypsin and glucose-6-phosphatase (G6P), and secretion of alpha-fetoprotein (AFP) and albumin (ALB). They found that ALB and G6P were detected earlier and higher levels of urea and AFP were produced in the 3D culture compared to those in the 2D culture. Lee et al. [12] reported that DPSC spheres created by ultra-low attachment (ULA) culture plates possess a greater multilineage differentiation capacity compared to that in monolayer DPSCs, suggesting that a 3D culture probably better reflects the in vivo microenvironment of stem cells.

The aim of this study was to compare two microsphere-forming culture methods with the monolayer culture method in terms of cell viability and differentiation pattern in vitro. We examined the morphology, cell viability, and functional differentiation potential of DPSCs cultured in two different microsphere-forming 3D culture plates and analyzed their differentially expressed gene (DEG) profiles. 

## 2. Experimental Section

### 2.1. Cell Culture

Primary human DPSCs were purchased from Cell Engineering for Origin (CEFO Co. Ltd., Seoul, South Korea). DPSCs expressed the following cell-surface protein profile assessed using flow cytometry and polymerase chain reaction (PCR): CD 105(+), STRO-1(+) and Nestin-1(+). DPSCs were cultured in Dulbecco’s Modified Eagle’s Medium (DMEM, Corning, NY, USA) containing 10% fetal bovine serum (FBS), 2% antibiotics (1% penicillin and streptomycin), and 1% L-glutamine (Sigma Chemical Co., St. Louis, MO, USA) at 37 °C with 5% CO_2_, and the medium was replaced every 2 days. The cells were subcultured on reaching confluence and were used at passages 2–4. 

**Figure 1:**
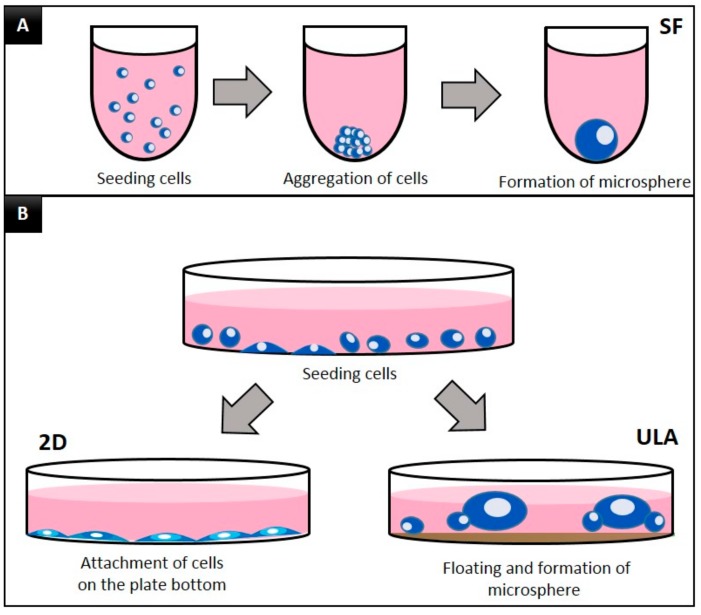
Schematic diagram of the experimental groups. (**A**) SF group: U-bottomed StemFit 3D (SF) plates, (**B**) two-dimensional (2D) group, conventional flat-bottom cell culture plate, ULA group: ultra-low attachment plate.

A schematic diagram of the experimental groups is presented in Figure 1. For the 2D monolayer culture (2D group), DPSCs were subcultured in a 60π plate. DPSCs were trypsinized using 0.25% Trypsin-Ethylenediaminetetraacetic acid (EDTA; Gibco, Grand Islands, NY, USA) for 3 min. After treatment with trypsin, the DPSCs were transferred to a 15 mL conical tube and centrifuged at 1500 rpm for 3 min and were then subcultured in a 60π 2D plate at a seeding density of 0.4 × 10^6^ cells/well.

For the 3D spheroid culture, Corning^®^ ultra-low attachment plates (ULA, Corning, NY, USA), which are flat bottomed plates, and Prosys^®^ StemFit 3D (SF, Prodizen Inc., Seoul, Korea), which are U-bottom plates, were used. For these 3D groups, the cell plating procedure was followed in the same manner as for the 2D group until the centrifugation step. Following the manufacturer’s recommendations, the DPSCs were subcultured at a seeding density of 0.4 × 10^6^ cells/well for the ULA group and 1.2 × 10^6^ cells/well for the SF group. Cells in all groups were cultured for 7 days. DPSC morphology was observed daily using a fluorescence microscope (JuLi, NanoEntek, Seoul, Korea).

### 2.2. Cell Proliferation Assay

Viability of the cells in each group was evaluated both quantitatively and qualitatively on days 1, 3, 5, and 7. Cell proliferation was determined using the cell counting kit-8 (CCK-8, Dojindo, Tokyo, Japan) assay by following the manufacturer’s protocol. Cells in the SF and ULA groups were transferred to a 15 mL conical tube and centrifuged at 1500 rpm for 3 min. DPSCs were then transferred to a 60π 2D plate and cultured for 12 h to allow the DPSCs to attach to the plate. Next, 2.2 mL of a 1:10 solution of CCK-8 and medium were added and incubated for 4 h. The solution was divided into 20 wells of a 96-well plate (110 µL/well), followed by measuring the absorbance. The optical density (OD) value was measured at 450 nm for 5 s. Readings from three parallel wells were averaged for each group.

A cell viability Live/Dead kit (Invitrogen, Ltd, Paisley, UK) assay was also performed to qualitatively evaluate the viability of the DPSCs. Ethidium homodimer (EthD-1), calcein AM, and Dulbecco’s phosphate-buffered saline (DPBS) were mixed at a ratio of 2:1:1000 to prepare a reagent mix. This reagent mix was added and incubated with the cells for 5 min and the fluorescence was detected using a fluorescence microscope (IX71-F32PH, Olympus, Tokyo, Japan). 

### 2.3. In Vitro Functional Multilineage Differentiation

DPSCs were induced to differentiate in adipogenic, osteogenic, or chondrogenic differentiation media. For each group, control cultures were maintained in media without induction of differentiation. 

To induce osteogenic differentiation, the cells were plated in basal medium (DMEM with 5% FBS, 2% antibiotics) at the appropriate confluence (2D and ULA: 1.5 × 10^5^, SF: 1.2 × 10^6^). Cells were incubated for 48 h, after which the medium was changed to osteogenic supplementation medium containing dexamethasone (10 nM/L), L-ascorbic acid (100 µM/L), and β-glycerophosphate (10 mM/L). The medium was replaced with differentiation medium every 2–3 days, and the cells were incubated for up to 20 days. To evaluate the extent of mineralization, the cells subjected to osteogenic induction were washed with PBS, fixed in 70% ethanol for 15 min, rinsed with distilled water, and stained for 3 min with Alizarin Red S (20 mM, pH 4.2; Sigma). The cultures were rinsed five times with distilled water. Thereafter, PBS was added, and microscopic images were taken.

To induce adipogenic and chondrogenic differentiation, the cells were plated and grown in adipogenic and chondrogenic differentiation-inducing media (STEMPRO Adipogenesis Differentiation Kit, STEMPRO Chondrogenesis Differentiation Kit; Gibco, Grand Island, NY, USA), and the DPSCs were grown for 28 and 14 days, respectively. The extent of adipogenic differentiation was assessed by staining the cells with Oil Red O on the 28th day. Cells were then washed with PBS two times, fixed with 4% paraformaldehyde for 15 min, and washed with distilled water. Next, the cells were stained with 0.25% Oil Red O solution for 20 min and rinsed five times with distilled water. The extent of chondrogenic differentiation was assessed by staining the cells with 1% Alcian blue for 3 min, followed by rinsing five times with distilled water. Next, PBS was added, and microscopic images were taken.

### 2.4. RNA Isolation and Sequencing 

Total RNA was isolated using TRIzol reagent (Invitrogen). RNA quality was assessed in the Agilent 2100 bioanalyzer using the RNA 6000 Nano Chip (Agilent Technologies, Amstelveen, The Netherlands), and RNA quantification was performed using an ND-2000 Spectrophotometer (Thermo Inc., Wilmington, DE, USA).

An RNA-sequencing (RNA-seq) library was generated using QuantSeq 3′ mRNA-Seq Library Prep Kit (Lexogen, Inc., Vienna, Austria) according to the manufacturer’s instructions. In brief, 500 ng of total RNA was prepared and an oligo-dT primer containing an Illumina-compatible sequence at its 5′ end was hybridized to the RNA and reverse transcription was performed. After degradation of the RNA template, second strand synthesis was initiated using a random primer containing an Illumina-compatible linker sequence at its 5′ end. The double-stranded library was purified using magnetic beads to remove all reaction components and amplified to add the complete adapter sequences required for cluster generation. The finished library was purified from the PCR components. High-throughput sequencing was performed as single-end 75 sequencing using NextSeq 500 (Illumina, Inc., San Diego, CA, USA). 

To annotate gene expression, QuantSeq 3′mRNA-Seq reads were aligned using Bowtie2 [13]. Bowtie2 indices were either generated from genome assembly sequences or representative transcript sequences for aligning to the genome and transcriptome. The alignment file was used for assembling transcripts, estimating their abundance, and detecting differential expression of genes. DEGs were determined based on the counts from unique and multiple alignments using coverage in Bedtools [14]. The read count (RC) data were processed based on the quantile normalization method using Edge R within R using Bioconductor [15]. Gene classification was based on searches performed by DAVID (http://david.abcc.ncifcrf.gov/) and Medline databases (http://www.ncbi.nlm.nih.gov/).

### 2.5. Statistical Analysis

Statistically significant differences in the DPSC cell viability data were determined using one-way analysis of variance (ANOVA) and Bonferroni tests in SPSS 23 (Statistical Package for Social Science, version 23.0, IBM Corporation, Chicago, IL, USA). Two-way ANOVA was performed to determine the interaction between “culturing method” and “culturing time.” Statistical significance was set at a confidence level of 95%, and *p* < 0.05 was considered statistically significant.

## 3. Results

### 3.1. Morphology

**Figure 2:**
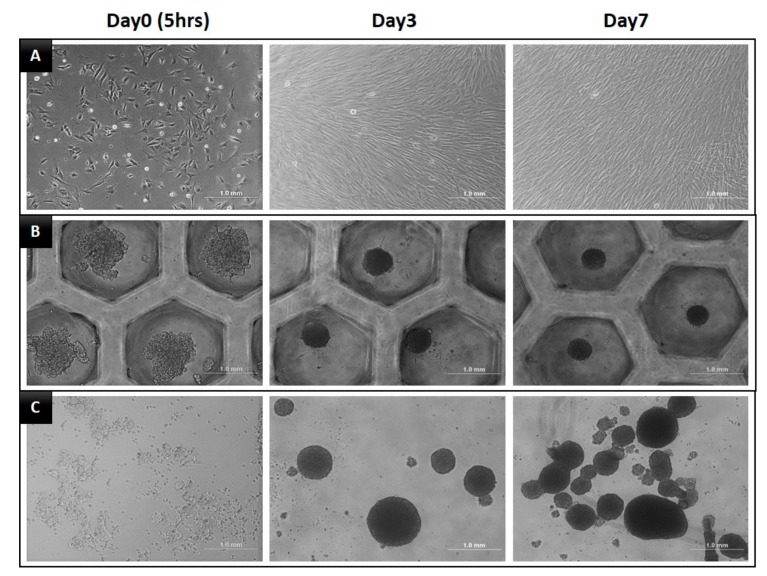
Morphology of DPSCs during seven days of incubation in three different culture plates. (**A**) 2D group, with plain culture plate, (**B**) SF group, with U-bottom plate, and (**C**) ULA group, with flat-bottom ultra-low attachment plate.

The daily microscopic observations of DPSCs cultured in the 2D, SF, and ULA plates are shown in Figure 2. The cells showed a fibroblastic morphology when cultured in 2D. The SF group showed a spherical form and the ULA group showed a free form mass. The number of DPSCs in the 2D group increased with time, while the size of the spheroids in the SF group decreased and aggregated densely with time. 

### 3.2. Cell Viability

**Figure 3:**
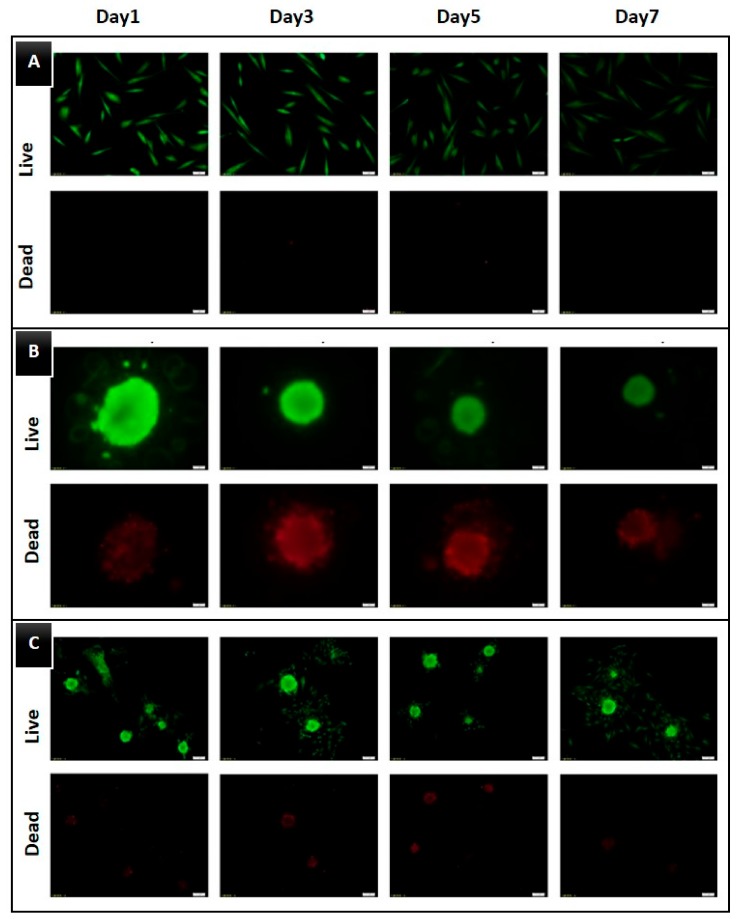
Live/Dead cell assay images of each group on days 1, 3, 5, and 7. (**A**) 2D group, with plain culture plate, (**B**) SF group, with U-bottom plate, (**C**) ULA group, with flat-bottom ultra-low attachment plate. The live and dead cell images are shown.

The results of the Live/Dead assay over a period of seven days are shown in Figure 3. In the 2D group, almost all the DPSCs were alive and dead cells were rarely observed. In the 3D groups, the majority of DPSCs were alive and displayed green fluorescence. However, both SF and ULA groups presented a slightly increased number of dead cells from day three, compared to that in the 2D group.

The results of the cell viability test using the CCK-8 assay and the statistical analysis of the data using two-way ANOVA are presented in Table 1 and Table 2. The absorbance of the 3D group showed a decreasing pattern, in contrast to the increasing pattern in the 2D group. In the SF and ULA groups, the absorbance continued to decrease significantly until the 5th day (*p* < 0.05). Two-way ANOVA revealed that the factors, “culturing time” and “culturing method”, significantly affected the absorbance (*p* < 0.05). 

### 3.3. Multilineage Differentiation Capacity

**Figure 4:**
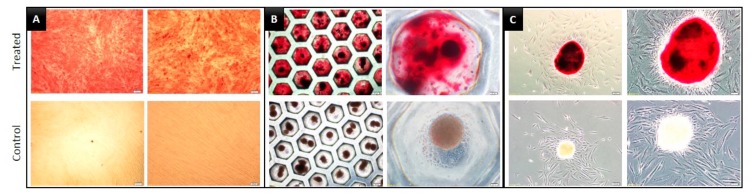
Images of dental pulp stem cells (DPSCs) induced to undergo osteogenic differentiation for 20 days and stained with Alizarin Red S. (**A**) 2D, (**B**) SF, and (**C**) ULA group, respectively.

**Figure 5:**
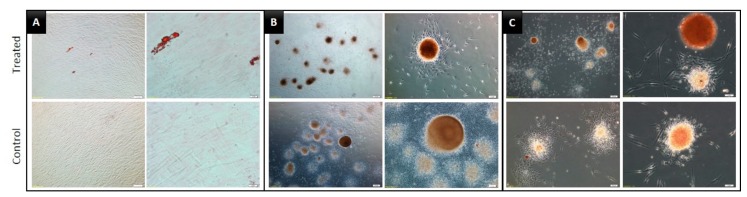
Images of DPSCs induced to undergo adipogenic differentiation for 28 days and stained with Oil Red O. (**A**) 2D, (**B**) SF, and (**C**) ULA group, respectively.

**Figure 6:**
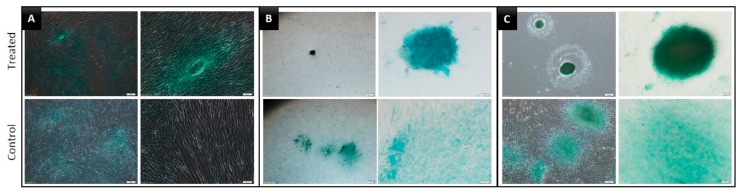
Images of DPSCs induced to undergo chondrogenic differentiation for 14 days and stained with Alcian blue. (**A**) 2D, (**B**) SF, and (**C**) ULA group, respectively.

We evaluated the multilineage functional differentiation capacity of DPSCs by culturing them in osteogenic, adipogenic, and chondrogenic induction media. In the induction of osteogenic differentiation media, all the experimental groups presented relatively higher staining with Alizarin Red S in the treated groups, compared to that in the respective control groups. However, compared to that in the 2D group, both the 3D groups, SF, and ULA, presented much greater osteo/odontogenic differentiation, as confirmed by Alizarin Red staining (Figure 4). Oil Red O and Alcian blue staining were clearly visible in the SF and ULA groups, indicating adipogenic and chondrogenic differentiation, respectively. However, staining intensity was low in the 2D group (Figure 5 and Figure 6).

### 3.4. Analysis of Gene Ontology and Gene Expression Profile 

**Figure 7:**
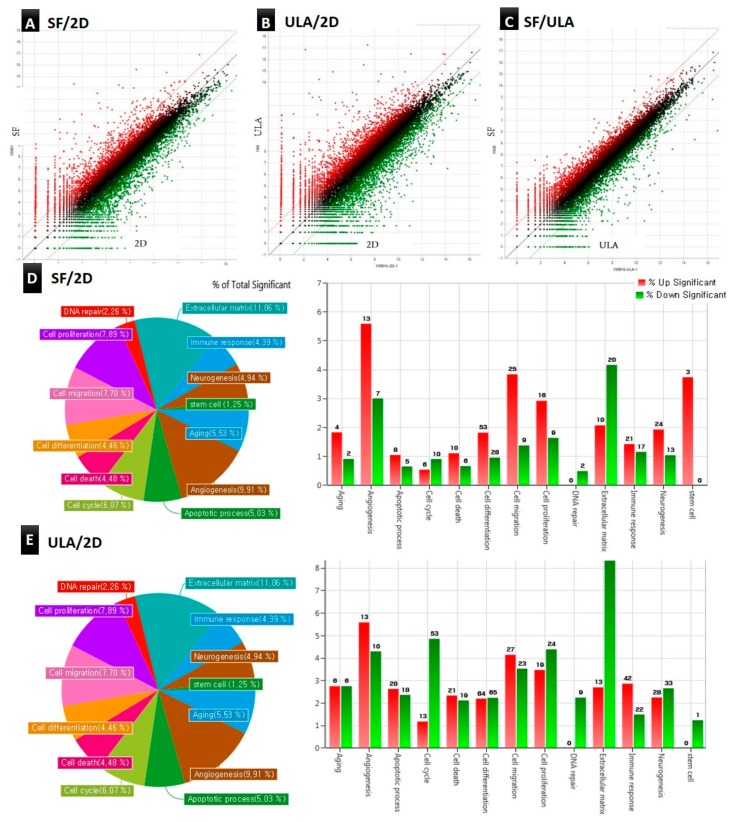
(**A**–**C**) Scatter plot of normalized data for two-fold threshold (*p* < 0.05). The differentially expressed genes (DEGs) are shown in green/red for down-/upregulation in control (2D), respectively. (**D**,**E**) Overview DEGs of DPSCs of SF and ULA groups compared to control (2D) group, respectively. The percentage of total significant DEGs are presented in the circular graphs. The gene category charts showed the DEG distributions of DPSCs with the following parameters: 10-fold changes, normalized data (log2) = 4, *p* < 0.05.

**Figure 8:**
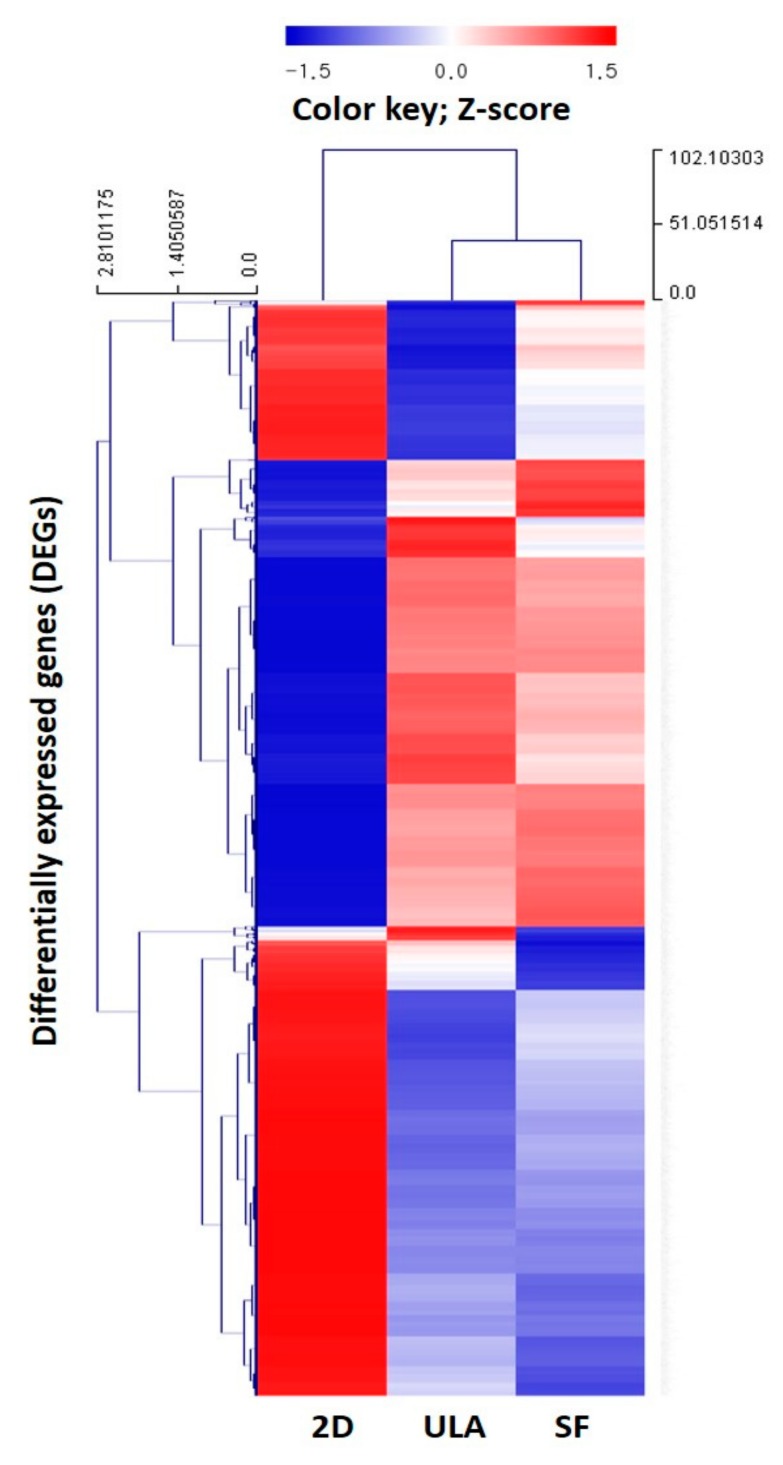
Heatmap illustrating Z-score normalized hierarchical clustering with dendrogram for three experimental groups. Transcripts with fold-change values larger than 2 with a *p* > 4, *p*-value < 0.05 were included in the analysis as differentially expressed genes (DEGs). The red and blue color indicate up- and down-expression of Z-score, respectively. Color density indicating the levels of fold changes are shown.

QuantSeq 3’mRNA-seq was used to compare multiple gene expression profiles of the 2D, SF, and ULA groups. A total of 25,737 DEGs in response to the experimental groups were identified, and detailed information of the top 10 upregulated genes in expression by RNA-seq were listed in Table 3. To investigate the biological functions and characteristics of the 25,737 DEGs, the expressed data were organized into gene ontology (GO) terms analysis. We observed that the significantly overrepresented GO terms (*p* < 0.05) included the gene categories related to aging, angiogenesis, apoptotic process, cell cycle, cell death, cell differentiation, cell migration, cell proliferation, DNA repair, extracellular matrix, immune response, neurogenesis, and stem cells. Figure 7A–C shows scatter plots of the comparative expressions in the groups. The scatter plot shows a highly wide distribution pattern for SF/2D and ULA/2D, but a close distribution (to straight line) for SF/ULA. Hierarchical clustering analysis was performed to display the DEG expression patterns. The results were corresponded with the data of the heatmap with dendrogram (Figure 8), which revealed the genetic distance of the 2D and microsphere-forming experimental groups (ULA and SF). Similar DEG regulation patterns between SF and ULA groups are presented; however, a comparatively different DEG regulation pattern between 2D and 3D groups is observed. Figure 7D,E shows an overview of the DEGs in DPSCs of SF and ULA groups compared to the 2D group. We found that the overexpressed gene categories in the SF group compared to the 2D group, included angiogenesis (8.62% significant DEG expression; number of up-/downregulated gene counts are 13/7), cell migration (5.24%; 25/9) cell differentiation (2.80%; 53/28), cell proliferation (4.59%; 16/9), and stem cell (3.75%; 3/0). In the ULA group compared to the 2D group, the overexpressed gene categories included angiogenesis (9.91%; 13/10), cell migration (7.70%; 27/23), cell differentiation (4.46%; 64/65), and immune response (4.39%; 42/22). 

## 4. Discussion

DPSCs have been investigated in several studies owing to their potential application in tissue engineering, easy availability, and versatility. DPSCs express mesenchymal markers such as CD29, CD44, CD59, CD73, CD90, and CD146, and do not express hematopoietic markers such as CD34, CD45, and CD11b. Thus, they show immense potential in the field of regenerative medicine [16]. Alge et al. compared DPSCs and bone marrow mesenchymal stem cells (BMMSCs) with respect to several parameters including proliferation rate, colony formation, clonogenic potential, and mineralization potential. The results revealed that DPSCs have a higher proliferation rate, greater clonogenic potential, higher population of stem/progenitor cells, and may also have increased mineralization potential compared to that of the BMMSCs [17]. 

When the DPSCs were grown in 2D cultures, fibroblastic morphology was observed, which has been mentioned in several previous studies. The spontaneous spheroid formation technique, which is used in this study, has some limitations. In this method, it is difficult to control the size and composition of the spheroids, create spheroids with a small number of cells, and set up the right ratio of two different cell types in spheroids when performing co-cultures [10]. However, in the SF group, one or sometimes two DPSC spheroids were observed in each small well, which, to some extent, complemented some limitations of the spontaneous spheroid formation technique. However, the ULA group showed a free-floating DPSC mass in the ULA plate. Based on these observations, the first null hypothesis was rejected.

The StemFit 3D plate consists of numerous small wells, making it easy to change media compared to that in the ULA plate. The ULA group carries a risk of cell loss during media suction because the cells float freely in the plate. Therefore, during media change, all the cells were harvested and centrifuged, and then the media above was suctioned leaving the dense cells below. This process was time-consuming; however, the process for the SF group was easier, and if suctioned carefully, it was possible to remove only the media because each well prevented cells from being lost. The ability to control the size and shape of the spheroid was an added advantage of the StemFit 3D plates.

The results of the proliferation assay, observed using optical microscopic imaging, showed that the number of cells in the 3D groups did not increase with time, whereas there was an increase in the cell number in the 2D group. In contrast, the spheroid collapsed, and its size decreased. The CCK-8 results also showed similar results: the absorbance of the control group increased steadily, whereas that of the 3D groups decreased with time. In the Live/Dead assay, dead cells were rarely found in the control group, whereas in 3D groups, dead cells were observed to be gradually arising from the center of the spheroid. Although DPSCs are adherent-type cells, they appeared to make spheroids even without scaffolds. Additionally, dead cells appeared to arise from the center of the spheroids is probably because of the insufficient supply of nutrition to the central part. In regenerative dentistry, the development of new scaffolds for regenerative endodontics is an important new field of dental materials research [18]. Particularly, hydrogels have been extensively studied as tissue engineering scaffolds because of their favorable biological properties [19]. In a recent study, Cavalcanti et al. assessed the compatibility of Puramatrix with DPSC growth and differentiation. They demonstrated that after 21 days in tooth slices containing Puramatrix, the DPSCs expressed DMP-1 and dentin sialophosphoprotein, putative markers of odontoblastic differentiation, representing a promising new alternative of injectable scaffolds for dental tissue engineering [20]. Although scaffolds have many advantages, the possibility of inflammation due to the presence of a scaffold has also been reported. Therefore, in this study, a scaffold-free method was used [21]. 

Despite severe culture conditions, odontogenic differentiation could be observed in the 3D groups. However, mild Alizarin Red S staining was observed on the 10th day and relatively strong staining was visible on the 20th day in the control groups as well. It was difficult to discern whether this was due to an error in the experimental setup or if it was an authentic odontogenic differentiation. Nevertheless, it was difficult to conclusively state that odontogenic differentiation was promoted more by the 3D culture than the 2D because only a qualitative evaluation was performed and there was no quantitative assessment. However, because the intensity of the Alizarin Red S stain was more in the 3D groups and a clear difference was observed on the 10th day, and these results corresponded to a recent previous investigation [1], which showed that the 3D spheroid-forming culture condition promotes the cell-to-cell, and cell-to-extracellular matrix interactions, leading to higher induction of odonto/osteoblastic differentiation. Unlike monolayer 2D culture, DPSCs in 3D culture dishes formed the microspheroids floating in the culture plates. Due to the limitation of staining methodology, there were several losses of spheroids during the staining procedure, the quantitative comparison with the 2D group were expected to present incorrect outcomes with many biases. In addition, the quantitative examination seemed not desirable because the number of cells seeding themselves differs between 2D and 3D groups. To overcome these limitations, we only presented the microscopic images of stained microspheroids (Figure 4, Figure 5 and Figure 6) and analyzed the gene expression profiles by RNA sequencing. 

The development of high-throughput next-generation sequencing (NGS) has allowed for profiling the transcriptomics via enabling RNA analysis through the sequencing of complementary DNA [22]. Our RNA-seq data demonstrated that a total of 25,737 DEGs were identified between three groups, and the top-10 upregulated genes of comparison between SF and ULA groups with the 2D group were listed in Table 3. Since the number of DEGs were so large, the analysis with the GO categories seemed more valuable. Figure 7D,E showed the overview of the comparison of DEG between 2D and 3D groups. The bar graph implicated the percentage of total significance, which were presented in the circular graph, so that the height of bar graphs corresponded to the order of significant GO categories. Also, hierarchical clustering with the dendrogram in Figure 8 indicated a closer correlation between ULA and SF groups, which were cultured in the microspheroid-forming culture dishes, whereas 2D was observed to be more distant from the two groups. 

## 5. Conclusions

Despite its limitations, our study demonstrated that DPSCs cultured in 2D and microsphere-forming plate culture methods presented different proliferation and differentiation properties. DPSCs cultured in the microsphere-forming plate showed increased multilineage differentiation capacities, which includes osteogenic, adipogenic, and chondrogenic differentiation. We also demonstrated that the DEG patterns were quite similar for two the 3D microsphere-forming culture methods. From the clinical point of view, the application of biodegradable scaffolds substantially implicates some issues, such as inflammation possibility, immune rejection, or xenogeneic infection. Thus, transplantation of stem cells without scaffold, such as 3D stem cell spheroids, can be an alternative option for tissue engineering and regenerative medicine, and further studies are needed for the clinical application of the microsphere system. The results of this study suggest that the DPSC microsphere can serve as a viable option for tissue engineering in regenerative endodontics.

## Figures and Tables

**Table 1:** Cell viability test by the cell counting kit-8 (CCK-8).

Group	Day 1	Day 3	Day 5	Day 7
2D	100.00 ± 3.71 Aa	100.66 ± 11.85 Aa	110.15 ± 1.38 Ab	120.56 ± 3.76 Ac
SF	100.00 ± 3.23 Aa	55.81 ± 1.27 Bb	51.40 ± 1.66 Bc	47.00 ± 0.95 Bd
ULA	100.00 ± 3.79 Aa	52.53 ± 1.08 Bb	33.76 ± 0.57 Cc	35.15 ± 0.67 Cc

Values are written as mean ± standard deviation. Within each column and row, the values with different capital and lowercase letters represent statistically significant differences (*p* < 0.05).

**Table 2:** Results of two-way analysis of variance of CCK-8.

	Type III Sum of Squares	df	F	Significance
Culturing Method	127,783.6	2	3907.67	0.000
Culturing Time	48,252.2	3	983.71	0.000
Method × Time	50,905.9	6	518.91	0.000

df: degrees of freedom, F: F-value. The results were statistically analyzed using one-way analysis of variance (ANOVA) with a Bonferroni post-hoc comparison. A two-way ANOVA was performed to determine the interaction between the culturing method and culturing (*p* < 0.05).

**Table 3:** List of the top 10 upregulated genes in expression by RNA-sequencing.

Gene	Description	Fold Changes	Related Biological Function
ULA/2D			
PTGS2	prostaglandin-endoperoxide synthase 2	930.52	Angiogenesis, cell differentiation, inflammatory response, cellular response to hypoxia, response to oxidative stress
AREG	amphiregulin	686.75	Cell differentiation, cell proliferation, cell-cell signaling
EREG	epiregulin	271.40	Cell differentiation, cell proliferation, angiogenesis, cell cycle, cell-cell signaling, mRNA transcription
GFAP	glial fibrillary acidic protein	54.93	Extracellular matrix organization, regulation of protein complex assembly, response to wound healing
NEFM	neurofilament, medium poly peptide	54.31	Neurofilament bundle assembly, axon development
NR4A3	nuclear receptor subfamily 4 group A member 3	46.59	Apoptotic process, cell proliferation, regulation of transcription,
MYCN	N-myc proto-oncogene protein	35.81	Regulation of transcription, cell proliferation, cell differentiation, cell death regulation
SOD2	superoxide dismutase 2, mitochondrial	29.84	Apoptotic process, regulation of transcription, oxidation-reduction process
ZC3H12A	zinc finger CCCH-type containing 12A	20.11	Cell differentiation, cell death, autophagy, p38MAPK cascade
LIF	leukemia inhibitory factor	18.79	Cell proliferation, cell differentiation
SF/2D			
AREG	amphiregulin	2066.98	Cell differentiation, cell proliferation
RANBP3L	RAN binding protein 3 like	500.44	Cell cycle, cell differentiation
MYCN	N-myc proto-onco gene protein	116.69	Regulation of transcription, cell proliferation, cell differentiation, cell death regulation
EREG	epiregulin	111.00	Angiogenesis, cell cycle, cell differentiation, cell proliferation
NKD1	naked cuticle homolog 1	107.20	DNA repair, Wnt signaling pathway,
PTGS2	prostaglandin-endoperoxide synthase 2	97.83	Angiogenesis, cell differentiation, inflammatory response
CNTN4	contactin 4	36.70	Cell adhesion, cell differentiation, axonogenesis,
GFAP	glial fibrillary acidic protein	21.71	Extracellular matrix organization, regulation of protein complex assembly, response to wound healing
FGFR3	fibroblast growth factor receptor 3	20.27	Bone mineralization, cell-cell signaling, apoptotic process, cell proliferation, cell differentiation,
NR4A3	nuclear receptor subfamily 4 group A member 3	18.71	Apoptotic process, regulation of transcription, cell proliferation
